# 

**Published:** 1887-08

**Authors:** 


CONTENTS.

COMMUNICATIONS.

Address on Dental and Oral Surgery .......... 369
Address by Dr. Kate C. Moody .......... 378
Recent Advances in Preventative Medicine .......... 388
Address by Dr. J. T. McMilen .......... 392

SELECTIONS.

Incubation and Transmission of Epidemic Parotitis .......... 395
Cocaine as a Local Anæsthetic .......... 395
On the Value of the Hemorrhage in the Treatment of Wounds .......... 396
The Influence of Disease upon Population .......... 397
Druggists' Orders .......... 398
Give the Babies Water .......... 399
Hemoptysis—Error of Diagnosis .......... 399
An Apparatus for the Relief of Writers' Cramp .......... 400
Hygienic Handwriting .......... 402
Salivary Calculus .......... 402
A Story of Science .......... 403
Dentistry in Germany .......... 404
The Effects of Ether on Living Nerves .......... 404
Antiquity of Mercury .......... 405
Hygiene in Early Youth .......... 405

CORRESPONDENCE.

International Congress .......... 406

EDUCATIONAL.

University of Tennessee .......... 409
Central University of Kentucky .......... 410
Obituary .......... 410
Salmagundi .......... 411
How to give Iron .......... 414
Acids in Digestion .......... 414

EDITORIAL.

Ninth International Medical Congress .......... 415

Registration Blank .......... 415

International Congress .......... 417

